# Dissecting Loss of Heterozygosity (LOH) in Neurofibromatosis Type 1-Associated Neurofibromas: Importance of Copy Neutral LOH

**DOI:** 10.1002/humu.21387

**Published:** 2010-10-28

**Authors:** Carles Garcia-Linares, Juana Fernández-Rodríguez, Ernest Terribas, Jaume Mercadé, Eva Pros, Llúcia Benito, Yolanda Benavente, Gabriel Capellà, Anna Ravella, Ignacio Blanco, Hildegard Kehrer-Sawatzki, Conxi Lázaro, Eduard Serra

**Affiliations:** 1 Institut de Medicina Predictiva i Personalitzada del Càncer (IMPPC)Badalona, Barcelona, Spain; 2 Departament de Genètica, Institut d'Investigació Biomèdica de Bellvitge (IDIBELL), L'Hospitalet de LlobregatBarcelona, Spain; 3 Programa de Diagnòstic Molecular de Càncer Hereditari, Laboratori de Recerca Translacional, Institut Català d'Oncologia (ICO)—IDIBELL, L'Hospitalet de LlobregatBarcelona, Spain; 4 Genes and Cancer Group, Programa d'Epigenètica i Biologia del Cáncer (PEBC), IDIBELL, Hospital Duran i Reynals, L'Hospitalet de LlobregatBarcelona, Spain; 5 Programa de Consell Genètic en Càncer, ICO-IDIBELL, L'Hospitalet de LlobregatBarcelona, Spain; 6 Unit of Infections and Cancer (UNIC), Cancer Epidemiology Research Programme, ICO—IDIBELL, CIBERESP, L'Hospitalet de LlobregatBarcelona, Spain; 7 Servei de Dermatologia, Hospital de la Creu RojaBarcelona, Spain; 8 Institute of Human Genetics, University of UlmUlm, Germany

**Keywords:** NF1, neurofibroma, LOH, mitotic recombination, variation, modifier genes

## Abstract

Dermal neurofibromas (dNFs) are benign tumors of the peripheral nervous system typically associated with Neurofibromatosis type 1 (NF1) patients. Genes controlling the integrity of the DNA are likely to influence the number of neurofibromas developed because dNFs are caused by somatic mutational inactivation of the *NF1* gene, frequently evidenced by loss of heterozygosity (LOH). We performed a comprehensive analysis of the prevalence and mechanisms of LOH in dNFs. Our study included 518 dNFs from 113 patients. LOH was detected in 25% of the dNFs (*N* = 129). The most frequent mechanism causing LOH was mitotic recombination, which was observed in 62% of LOH-tumors (*N* = 80), and which does not reduce the number of *NF1* gene copies. All events were generated by a single crossover located between the centromere and the *NF1* gene, resulting in isodisomy of 17q. LOH due to the loss of the *NF1* gene accounted for a 38% of dNFs with LOH (*N* = 49), with deletions ranging in size from ∼80 kb to ∼8 Mb within 17q. In one tumor we identified the first example of a neurofibroma-associated second-hit type-2 *NF1* deletion. Analysis of the prevalence of mechanisms causing LOH in dNFs in individual patients (possibly under genetic control) will elucidate whether there exist interindividual variation. Hum Mutat 32:78–90, 2011. © 2010 Wiley-Liss, Inc.

## Introduction

One of the main challenges in the study of Neurofibromatosis type 1 (NF1, MIM♯ 162200) is to have the ability to predict the course of the disease. Prognostic markers can only be found if we have a complete understanding of how the genetics and the environment influence the different traits that compose the NF1 phenotype. One such trait is the development of dermal neurofibromas (dNFs) in multiple numbers, benign tumors of the peripheral nervous system.

The number of dNFs developed in NF1 patients is highly variable, ranging from tens to thousands of tumors in a single patient. The number of neurofibromas increases throughout life, and their growth is highly influenced by the hormonal status of the patient. Different studies meant to clarify the heritability of NF1 traits have identified the existence of a strong genetic component influencing the variable number of dNFs developed, that seem to follow a polygenic model [Easton et al., 1993; Sabbagh et al., 2009]. The role of the first-hit mutation at the *NF1* locus (MIM♯ 613113; NG_009018.1) in this variation is still not clear. Two types of constitutional *NF1* mutations have been found to influence neurofibroma number: Type-1 deletions (1.4-Mb deletions with breakpoints located within *NF1*-REPs a and c) seem to be associated with the early onset of a large number of dNFs [Kayes et al., 1994; Wu et al., 1995] and the c.2970-2972 delAAT mutation has been identified in patients with an absence of dNFs [Upadhyaya et al., 2007]. However, these two types of constitutional mutations only account for a small percentage of NF1 patients. Different studies also found a strong correlation between first-, second-, and even third-degree relatives [Sabbagh et al., 2009; Szudek et al., 2002], suggesting a role of the *NF1* germline mutation. However, it has also been observed that patients bearing the same germline mutation, even affected patients from the same family, can exhibit a very different number of dNFs [Ars et al., 2000; Carey et al., 1979], although possible confounding factors, such as patient's age, need to be analyzed more carefully.

Neurofibromas are caused by the double inactivation of the *NF1* gene ([Sawada et al., 1996; Serra et al., 1997], and many other works reviewed in [De Raedt et al., 2008]). Neurofibromas are composed of different cell types, but only Schwann cells bear a double inactivation of the *NF1* gene [Kluwe et al., 1999; Maertens et al., 2006; Serra et al., 2000]. The ability to isolate pure populations of *NF1*(−/−) Schwann cells and analyze their genetic material has demonstrated that the majority of somatic *NF1* inactivations in dNFs are due to point mutations [Maertens et al., 2006]. Because somatic mutations and neurofibroma-genesis are causally linked, genes controlling DNA repair mechanisms are good candidates for being modifiers of the number of dNFs developed [Maertens et al., 2006; Serra et al., [Bibr b30][Bibr b33]]. Furthermore, it has been reported that in a significant percentage of dNFs (∼20%) somatic inactivation is evidenced by loss of heterozygosity (LOH) (reviewed in [De Raedt et al., 2008, Serra et al., 2007; Steinmann et al., 2009]), also including spinal neurofibromas [Upadhyaya et al., 2009].

It has been known for a long time that different mechanisms are responsible for LOH in tumors, such as mitotic recombination, mechanisms that cause deletions of genetic material, or the loss of a whole chromosome by nondisjunction with or without reduplication [Cavenee et al., 1983; Tischfield, 1997]. Despite the number of published articles describing the detection of somatic LOH in the *NF1* region in dNFs ([Colman et al., 1995] and reviewed in [De Raedt et al., 2008]), there have only been a few attempts to characterize the mechanisms underlying LOH in dNFs [De Raedt et al., 2006a; Serra et al., 2001b; Steinmann et al., 2009; Upadhyaya et al., 2008, 2009]. So far, a comprehensive study of several hundred dNFs is missing. This should include the evaluation of LOH frequency, the extent of the respective LOH regions, and the identification of the number of copies of the *NF1* gene. We performed such a study to investigate the mutation mechanisms involved in the LOH of the *NF1* gene in dNFs from 113 NF1 patients. These mechanisms are likely to be mainly regulated by genes, which in a second step could be analyzed as modifiers of the number of dNFs. We believe that having a clear picture of the frequency of LOH in dNFs and understanding the importance of the different mechanisms underlying *NF1*-LOH is necessary for any further attempt to identify genes influencing neurofibroma development.

## Material and Methods

### Patients and Neurofibromas

A total of 518 dermal neurofibromas (dNFs) from 113 Neurofibromatosis type 1 (NF1) patients were included in this study. The patients were diagnosed according to standard diagnostic criteria [DeBella et al., 2000]. All patients gave written informed consent for the molecular studies performed. 318 of the 518 neurofibromas analyzed were already investigated for the presence of LOH in a previous study [Serra et al., 2007]. Of these 318 tumors, 92 exhibited LOH. However, these 92 neurofibromas required a more exhaustive analysis than previously performed in order to determine the extent of LOH and the mutational mechanism involved. Therefore, these 92 dNFs were reanalyzed in this study using at least one of the techniques for detecting *NF1* copy number described below. In addition to these 92 tumors, 200 so far uncharacterized dNFs were included in the present study and investigated by the microsatellite multiplex PCR analysis. Those dNFs among the 200 that exhibited LOH were also analyzed using multiplex ligation-dependent probe amplification (MLPA) or paralog ratio analysis (PRA) techniques (see below), and in some of them (*N* = 19), SNP-array analysis was also performed. Neurofibromas were completely removed after minor surgery, undertaken by either a dermatologist or a surgeon. Surrounding nonneurofibroma tissue was removed and tumors were cut into different pieces. A piece of each tumor was preserved at − 80°C until DNA was extracted. Whenever possible, remaining pieces of each dNF were further cut, immersed in freezing solution [10% DMSO, 90% fetal bovine serum (FBS)], and preserved in liquid nitrogen until they were used to establish cell cultures.

### Statistical Analysis

Confidence intervals (CIs) shown in Table 1 were calculated using the Normal Approximation Method of the Binomial CI.

**Table 1:** Global View of LOH in Neurofibromas

No. of patients	113
dNFs analyzed	518
dNFs with LOH	129 (49 deletions/80 MRs)
% LOH tumors/total tumors	24.9
% LOH-deletion/total tumors (95% CI)	9.4 (6.8–11.9)
% LOH-recombination/total tumors (95% CI)	15.4 (12.3–18.5)
% LOH-deletion/LOH-tumors (95% CI)	38 (29.7–46.3)
% LOH-recombination/LOH-tumors (95% CI)	62 (53.7–70.3)

LOH, loss of heterozygosity; CI, confidence interval; dNF, dermal neurofibroma; MR, mitotic recombination.

Taking data published by different groups in the literature (see, i.e., [De Raedt et al., 2006a]), we assumed a general LOH prevalence in neurofibromas from NF1 patients of 20%. Because there is no information about the variation among NF1 patients regarding this prevalence, we assumed a constant prevalence of 20% in order to assess the minimal number of dNFs to be tested for each NF1 patient to estimate the LOH prevalence. As sampling fraction is large, the binomial approximation is no longer appropriate, and therefore we have used a finite population correction term to adjust the variance of the normal distribution to the more closely model of the hypergeometric density function [Freund, 1992]. Under different scenarios of number of dNFs per patient (see Supp. Fig. S1) we assessed that we need to analyze 10 dNFs to estimate the hypothesized prevalence with a precision of 20%. Increasing the number of neurofibromas analyzed would improve precision, but it has also to be taken into account the feasibility of analyzing a large number of neurofibromas from a single patient, both for the patient and for the research team.

### DNA Extraction

DNA from venous blood of the patients was isolated either by the Wizard Genomic DNA purification kit (Promega, Madison, WI) according to the manufacturer's instructions or by the “salting out” method as described elsewhere [Miller et al., 1988]. This DNA was used as control or reference DNA in the LOH analysis of tumors of the respective patients. Control DNA was also obtained in some cases from the skin of patients (or derived fibroblasts). DNA from skin, neurofibromas, Schwann cells, or fibroblast cultures was extracted using the Gentra Puregene Kit (Qiagen, Chatsworth, CA) following manufacturer's instructions. In all cases, purity and quality of DNA was assessed using a nanodrop spectrophotometer and gel electrophoresis analysis. All DNA samples were stored and preserved at 4°C and at − 80°C.

### LOH Analysis and Mutational Mechanism Characterization

In order to identify the type of somatic mutation generating LOH, all dNFs were investigated by an extended analysis of chromosome 17 microsatellites to determine LOH and its extent, and also by MLPA and PRA to characterize the *NF1* copy number. In some dNFs, the characterization of LOH breakpoints at high resolution and a global characterization of genome integrity were assessed by SNP-array analysis. All genomic analyses were performed using the NCBI36/hg18 assembly of the human genome.

### Microsatellite Multiplex PCR (MMP) Analysis

We have developed an MMP assay that allows the simultaneous amplification of 16 microsatellite markers (Supp. Table S1) in a single PCR reaction: 15 markers are located in chromosome 17 (2 markers in the p arm and 13 in the q arm) and 1 marker is located in the q arm of chromosome 2. Primers were dye-labeled with four different fluorophores, FAM (blue), NED (yellow), VIC (green), and PET (red). We designed PCR products of different sizes labeled with different colors in order to differentiate all of them in a single genotyping run. MMP reaction was performed using a Multiplex PCR kit from Qiagen in a 25-µl reaction with 100 ng of DNA as follows: an initial cycle of denaturation at 95°C for 15 min was followed by 25 cycles of denaturation at 94°C, annealing at 56°C and extension at 72°C, for 30 sec, 3 min, and 1.5 min, respectively, and by a final cycle of 60°C for 30 min. We checked that at 25 cycles the amplification reaction for all markers was still at exponential phase (data not shown). A total of 1 µl of PCR product was mixed with 9 µl of highly deionized formamide (Applied Biosystems, Bedford, MA) and 0.5 µl of 500 LIZ® Size Standard. PCR fragments were separated by capillary electrophoresis on an ABI 3130*xl* Genetic Analyzer. Peak height values were extracted using Peak Scanner software (Applied Biosystems). In our hands, using peak height values was more accurate than using peak areas, as shown by Paulson et al. [1999], and recommended by the instrument's manufacturer [Applied Biosystems, Gene Mapper Software, Loss of Heterozygosity (LOH) Analysis Getting Started Guide].

To assess the presence of LOH we performed an allelic imbalance analysis, described by the expression Q^LOH^ [Hahn et al., 1995] considering each microsatellite marker independently. Q^LOH^ is calculated by dividing the allele ratio (the ratio between the two alleles) of a given microsatellite from tumor DNA (tumor peak height allele 1/tumor peak height allele 2) by the allele ratio of the same microsatellite from control DNA (control peak height allele 1/control peak height allele 2) [Solomon et al., 1987]. For simplicity and comparative purposes we always have assigned peak height allele 1 to the allele showing LOH. LOH was always assessed by comparing pairs of control and tumor DNA from the same patient. We considered presence of LOH in neurofibromas when allele ratios comparing control and tumor samples (Q^LOH^) differed more than 20% (Q^LOH^<0.8).

### MLPA

We further analyzed the number of copies of the *NF1* gene present in DNA samples by using the MLPA technique designed by MRC-Holland. Because the company is improving the assay with time, different kits were used for the characterization of all dNFs: Salsa MLPA Kit P122 versions P122, P122-B1, and P122-C1. Briefly, this technique is based on a ligation-dependent probe amplification of 12 to 28 probes (depending on the kit used) encompassing the *NF1* area together with 11 to 15 additional control probes (also depending on the kit used). Probes differ in size by means of spacers of different length that are probe-specific. Only probes that are properly ligated can be amplified in the subsequent PCR reaction. Once amplified, PCR fragments are separated by capillary electrophoresis and peak intensities are analyzed. For the Salsa MLPA Kit P122, PCR products of different length (ranging from ∼100 bp to ∼400 bp, depending on the kit used) were separated on an ABI 3130*xl* Genetic Analyzer. Peak height values were extracted using Peak Scanner software (Applied Biosystems) and were normalized as described by Wimmer et al. [2006] for peak areas. Briefly, peak heights from individual probes were divided by the sum of all peak heights of a given sample, obtaining a relative value. Relative values of each probe were then divided by the mean of the relative values of that probe obtained only from control samples. After this normalization of peak heights, it was possible to analyze the *NF1* copy number. The criteria used to assess the number of copies of the *NF1* gene present was the following: a value between 0.8 and 1.2 indicated that two copies of the *NF1* gene were present; a value lower than 0.8 implied that only one copy of the *NF1* gene was present. In 13 cases, the presence of normal cells within neurofibromas bearing LOH compromised these criteria (see Supp. Fig. S2). For this reason in certain dNFs we had to assess *NF1* copy number using either the isolation of pure populations of neurofibroma-derived Schwann cells, performing again the MLPA technique in their isolated DNA, or by using only the Paralog Ratio Analaysis technique.

### PRA

This method was developed for the *NF1* region by De Raedt et al. [2006a] and determines the *NF1* copy number status of a sample DNA by comparing the peak height values of two PCR products of a single amplification reaction. This PCR reaction coamplifies a fragment of exon 22 present in the *NF1* gene and the same fragment bearing an insertion of 4 bp from a single *NF1* pseudogene located in chromosome 15. PRA reaction was performed in a 10-µl final reaction with 80ng of DNA as follows: an initial cycle of denaturation at 94°C for 3 min followed by 26 cycles of denaturation at 94°C, annealing at 55°C, and extension at 72°C for 30 sec each step. A final cycle of 72°C for 7 min was performed.

We used a dosage quotient analysis comparing the ratios between gene and pseudogene peaks obtained from a tumor and a control sample from the same patient (in triplicate) and established the presence of a deletion when the quotient of the ratios obtained were below 0.8, that is, when the differences between tumor and control DNAs were greater than 20%. With this technique only deletions covering the region of exon 22 from the *NF1* gene can be detected. In addition, it has to be taken into account that the *NF1* pseudogene located in chromosome 15 is placed in a copy number variation (CNV) region, and thus only control and tumor pairs from the same patient can be compared in the analysis, because both DNAs will contain the same number of copies in the CNV region.

### SNP-Array Analysis

To characterize LOH breakpoints of both deletions and mitotic recombinations, as well as to explore other possible gross alterations in the tumor genome, SNP-array analysis was performed in 19 dNFs, using the Illumina Infinium technology (Illumina, San Diego, CA). In all cases, pairs of control and neurofibroma DNA were analyzed. Twelve dNFs were analyzed using the Illumina 370-Quad beadchip (∼351,000 genotyping SNPs, ∼9,300 located in the 17th chromosome), and seven dNFs samples were investigated using the Human660W-Quad beadchip (∼592,000 genotyping SNPs, ∼15,600 located in the 17th chromosome). DNA used for this type of analysis was of the highest quality. DNA concentration, purity, and quality was first assessed using a nanodrop spectrophotometer and gel electrophoresis analysis. For an accurate quantification of DNA concentration, samples were further quantified using Quant-iT PicoGreen reagent (Invitrogen, Carlsbad, CA). Raw data were analyzed using the Illumina software GenomeStudio v2009.1 with the Genotyping v1.1.9 module. The GenomeStudio program provides two types of measurements. On the one hand, it provides a quantitative measurement of the B-allele frequency (BAF) that reflects the allelic imbalance of each SNP. On the other hand, the program generates the logR ratio metric, which reflects the number of DNA copies. The log*R* ratio is a log-transformed ratio of the measured SNP signal intensity by the expected intensity if two copies of DNA were present. To calculate the percentage of normal cells present in the tumor we used the same method as Assie et al. [2008]. After obtaining the BAF value of the LOH region, the percentage of cells exhibiting LOH was calculated using the appropriate equation depending on the somatic event generating LOH (deletion or recombination).

### REP-Mediated Deletion Analysis

**Figure 1:**
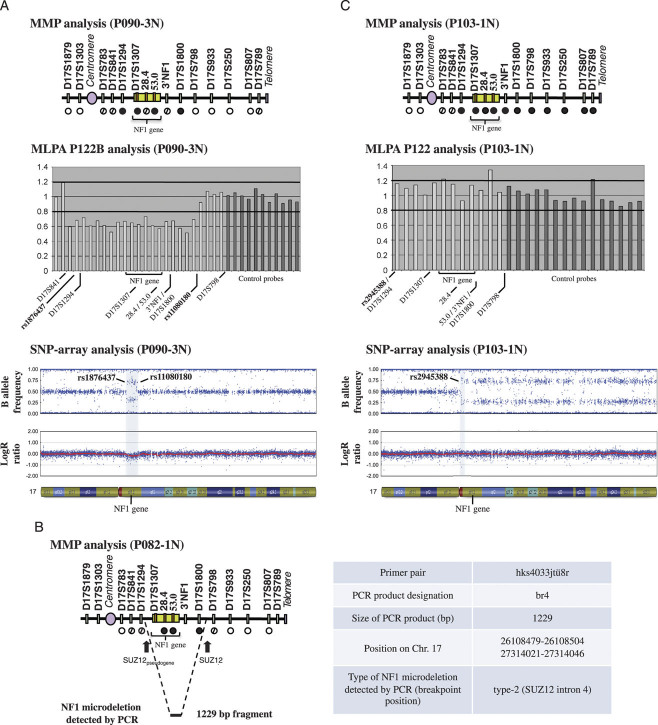
Mechanisms leading to LOH in neurofibromas. **A:** Mechanisms generating deletions. In all cases Microsatellite Multiplex PCR (MMP) analysis evidenced LOH in the *NF1* gene (NG_009018.1) and normally also in 5′ and 3′ regions adjacent to it. MLPA detected only one copy of the *NF1* gene. SNP-array analysis detected LOH (four-band pattern in the B allele frequency plot) and one copy of the *NF1* gene (LogR ratio <0). **B:** Nonallelic homologous recombination causing deletion. MMP detected LOH involving the *NF1* gene and adjacent regions, apparently not going further 5′ or 3′ of the *NF1*-REPs. A table summarizing the PCR conditions used for detecting the deletion breakpoint is depicted. Breakpoint localized in *SUZ12* intron 4. **C:** Homologous recombination. MMP evidenced LOH of almost all 17q chromosome. MLPA detected two copies of the *NF1* gene and the entire region analyzed. SNP-array detected LOH (four-band pattern in the B allele frequency plot) from the centromere to the end of the chromosome (17q telomere) and the presence of two copies (Log*R* ratio = 0) of the entire chromosome 17q. MMP (∘ = no LOH; dashed circles = noninformative, • = LOH). MLPA (Values between 0.8 and 1.2 = two copies. Values <0.8 = one copy). SNP-array: B allele frequency plot (0.5 = heterozygote; 0 or 1 = homozygote, other intermediate values = allelic imbalance); Log*R* ratio (0 = two copies; values <0 = one copy). [Color figures can be viewed in the online issue, which is available at wileyonlinelibrary.com.]

After performing Microsatellite Multiplex PCR Analysis and/or MLPA analysis, a few neurofibromas were suspicious of bearing deletions mediated by recombination between duplicated sequences in the *NF1* gene region (REP-mediated deletion). In these cases, we performed several diagnostic PCR reactions, as described by Steinmann et al. [2007], to identify somatic type-1 or type-2 *NF1* deletions with breakpoints either in the *NF1*-REPs or in the *SUZ12* sequences (see Fig. 1B).

### Neurofibroma-Derived Schwann Cell Culture

Neurofibroma-derived Schwann cells bearing somatic mutations (*NF1*−/−) were isolated as previously described [Serra et al., 2000]. Briefly, neurofibroma pieces that were preserved in liquid nitrogen were thawed and digested with collagenase and dispase (Worthington, Lakewood, NJ) for 18 hr at 37°C. Subsequently, tumors were mechanically dissociated and cell suspensions were plated in six-well cultured plates coated with poly-L-Lysine (Sigma, St. Louis, MO) and laminin (Invitrogen) in Schwann cell medium (SCM) and maintained at 37°C and 10% CO_2_. SCM is DMEM (Gibco, Grand Island, NY) with 10% FBS, 500 U/ml penicillin/streptomycin (Gibco), 0.5 mM 3-iso-butyl-1-methilxantine (IBMX, Sigma), 2.5 µg/ml insulin (Sigma), 10 nM β1-heregulin (R&D Systems, Minneapolis, MN), and 0.5 µM forskolin (Sigma). One day after plating, culture medium was replaced by SCM without forskolin, for 2–3 additional days. This process was repeated in cycles and cells were passaged as needed. Schwann cell purity was assessed in each passage by performing an S-100 staining as described [Serra et al., 2000]. Normally, after the third passage, cell cultures contained >95% Schwann cells.

## Results

### Mutational Mechanisms Leading to LOH in dNFs

Our goal is to have a large population of Neurofibromatosis type 1 (NF1) patients with a molecular phenotypic characterization. This consists of the estimation of LOH frequency implicated in the generation of dNFs and the elucidation of the mechanisms responsible for LOH. In doing so, we have obtained a clear picture of LOH in dNFs and the frequencies of the mechanisms responsible.

We have analyzed 518 dNFs obtained from 113 NF1 patients. First we developed a rapid and cost-effective methodology to obtain information on LOH presence and extent. Using this technique (MMP analysis) we identified 129 dNFs exhibiting LOH affecting the *NF1* gene (Supp. Table S2). In a second step of the analysis, we validated the presence of *NF1* copies by performing MLPA analysis. For many dNFs we also employed a PRA developed by De Raedt et al. [2006a] to determine *NF1* copy number (see Materials and Methods). Finally, we investigated *NF1* copy number, LOH extent, the percentage of cells bearing LOH and deletions or duplications in other genomic regions in 19 dNFs by genome-wide SNP-array analysis. Knowing the extent of the LOH and the number of copies of the *NF1* gene present in the tumor enabled us to identify the mutation mechanism responsible for each LOH detected (Table 1 and Supp. Table S2). dNFs are composed of distinct cell types (Schwann cells, fibroblasts, perineurial cells, etc.) in variable proportions, but only a group of Schwann cells carry the somatic *NF1* mutation. A high percentage of non-LOH cells within neurofibromas can greatly hamper the detection of a loss of 1 copy of the *NF1* gene using techniques such as MLPA or PRA. Therefore, we tested the limits of detection of MLPA and PRA techniques for correctly finding a deletion in the *NF1* gene in the context of different proportions of non-LOH cells within neurofibromas (Supp. Fig. S2). For doing so, we performed serial admixtures of two DNAs: one carrying a deletion in the *NF1* gene and the other bearing two intact copies of the gene, and analyzed these DNA admixtures using MMP, MLPA, and PRA techniques. We then evaluated the limits of detection of MLPA and PRA techniques for correctly finding an *NF1* deletion in relation to the Q^LOH^ values obtained using the MMP analysis in the different admixtures (Supp. Fig. S2 and Supp. Table S2). MLPA and PRA techniques were able to correctly detect an *NF1* deletion in neurofibromas with Q^LOH^ values below 0.58 for MLPA, and Q^LOH^ values below 0.76 for PRA (Supp. Fig. S2). None of the dNFs exhibiting LOH showed Q^LOH^ values above 0.74. We were not able to analyze the *NF1* copy number in 13 LOH-neurofibromas due to the lack of enough DNA material after the presence of LOH being detected (see Supp. Table S2). However, based on results obtained for the rest of LOH-tumors (*N* = 116) we assumed (and counted them as such) that for those exhibiting LOH in the whole 17q arm, LOH was caused by homologous recombination and for those with interstitial LOH, loss of heterozygosity was caused by deletion.

### LOH Affecting Copy Number

**Figure 2:**
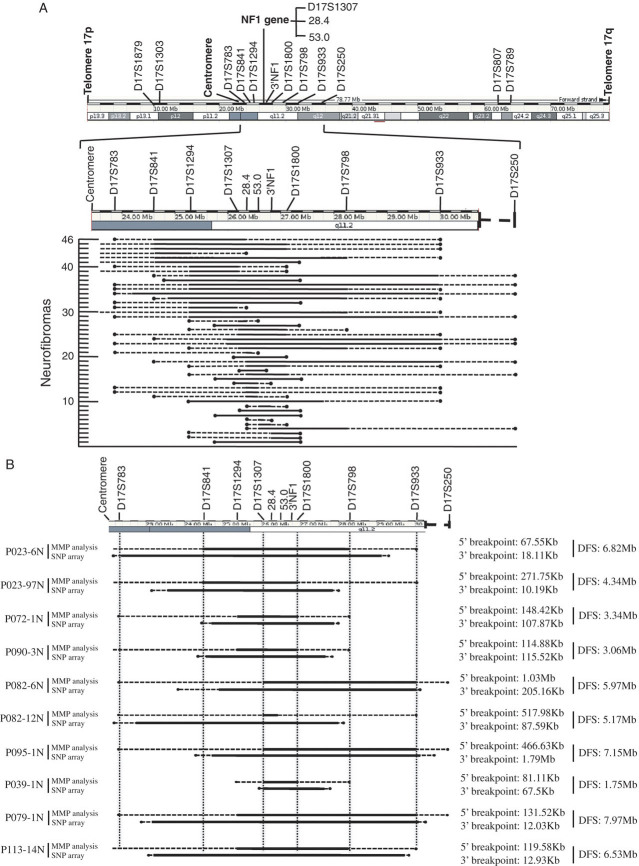
Deletion breakpoint analysis. **A:** Breakpoint analysis by Multiplex Microsatellite PCR (MMP) and by MLPA analysis. **B:** Deletion breakpoint mapping refinement by SNP-array. Solid black bar: deletion mapped by either MMP (A, B), by MLPA (A), or SNP-array (B); dashed line: uncertain region by either MMP (A, B) or SNP-array (B). Black circle: no deletion by either MMP (A, B), by MLPA (A) or SNP-array (B). DFS, Deletion fragment size. [Color figures can be viewed in the online issue, which is available at wileyonlinelibrary.com.]

We identified 49 dNFs that exhibited the loss of 1 copy of the *NF1* gene compared with their control tissue (see example in Fig. 1A, Table 1, and Supp. Table S2). LOH extension in all cases was affecting only the *NF1* gene or the *NF1* gene and adjacent genomic regions. The large distance between microsatellite markers and the lack of heterozygosity in some of them hampered a fine mapping of the precise extension of identified deletions. However, as is shown in Figure 2A, deletions expanded roughly from ∼80 kb to ∼8 Mb. The majority of interstitial deletions are presumed to be caused by DNA double-strand breaks (DBS) and subsequent illegitimate joining of DNA ends by homologous or nonhomologous end joining repair, or by homologous recombination repair [Bishop and Schiestl, 2003; Kohno and Yokota, 2006]. The structure of breakpoint junctions helps with the identification of the repair mechanism used, as the breakpoint can retain sequence signatures indicative of the mechanism involved in the repair. Characterizing breakpoints and understanding the exact mechanisms generating deletions was beyond the scope of this work. However, we used SNP-array analysis to study the integrity of the whole genome in 10 dNFs carrying deletions affecting the *NF1* gene, and in these cases we were also able to narrow down the deletion breakpoints (Fig. 2B). With this analysis we confirmed that deletions do not seem to involve DNA regions greater than ∼8 Mb. In the future it will be possible to further analyze and finally sequence the deletion breakpoints of these dNFs.

After performing MMP analysis and/or MLPA analysis, we observed that in two neurofibromas (P004-7N, P082-1N) the deletion breakpoints were located very close to the low-copy repeats (*NF1*-REPs and the *SUZ12* sequences) flanking the *NF1* gene region. In both patients a germline point mutation was already identified. To investigate whether the somatic deletions in these two tumors were mediated by nonallelic homologous recombination (NAHR) between these low-copy repeats, we performed PCR reactions [Steinmann et al., 2007] to detect the breakpoints of previously identified *NF1* microdeletions in patients with constitutional type-1 or mosaic type-2 deletions [De Raedt et al., 2006b; Steinmann et al., 2007]. We identified one dNF (P082-1N) (Fig. 1B) that carried a type-2 deletion. This type of *NF1* deletion is caused by nonallelic homologous recombination between the *SUZ12* gene and its pseudogene, which are located adjacent to the *NF1*-REPs. First-hit type-2 *NF1* microdeletions are identified in mosaic NF1 patients, and thus they are assumed to occur during early fetal development [Kehrer-Sawatzki and Cooper, 2008].

**Figure 3:**
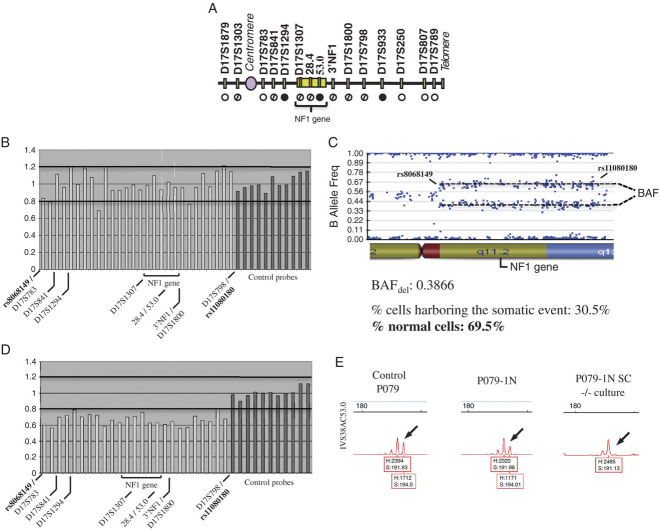
The presence of normal cells in neurofibromas affects the *NF1*-copy number detection by MLPA. **A:** MMP analysis of tumor P079-1N (see legend of Fig. 1 for nomenclature); **B:** MLPA of P079-1N detected two copies of the *NF1* gene; **C:** SNP-array analysis evidenced a high proportion (∼70%) of normal cells within tumor P079-1N; **D:** MLPA of P079-1N *NF1*(−/−) Schwann cell culture detected one copy of the *NF1* gene. **E:** Purity of the culture was evidenced by MMP analysis, comparing control, tumor, and SC culture. A total loss of one allele was detected in the SC culture. MLPA (values between 0.8 and 1.2 = two copies. Values <0.8 = one copy). [Color figures can be viewed in the online issue, which is available at wileyonlinelibrary.com.]

In 13 dNFs we detected LOH affecting only the *NF1* gene and flanking regions (Fig. 3A) (what in principle suggested the presence of an interstitial deletion) but when applying MLPA analysis, the same tumors were apparently exhibiting two copies of the *NF1* gene (Fig. 3B). These results opened up the possibility of new mechanisms causing LOH in dNFs, such as homologous recombination with two crossovers, one at 5′ and the other at 3′ of the *NF1* gene.

However, SNP-array analyses of several of these neurofibromas revealed that the percentage of non-LOH cells was quite high (Fig. 3C), an indication that was also supported by the high Q^LOH^ values obtained (e.g., Q^LOH^ = 0.62 for P079-1N, Supp. Table S2). In the cases where Q^LOH^ values were higher than 0.58, MLPA technique was not adequate for assessing the number of *NF1* copies (Supp. Fig. S2). Accordingly, we decided to perform additional analysis. For two neurofibromas we were able to culture pure populations of dNF-derived *NF1*(−/−) Schwann cells (Fig. 3E). In these two cases (P079-1N, P062-4N) when DNA isolated from pure *NF1*(−/−) Schwann cell populations was re-analyzed by MLPA, the loss of one copy of the *NF1* gene was detected (Fig. 3D). Thus, LOH detected in these two tumors was indeed caused by deletions. In 7 of the remaining 11 dNFs we were able to assess the presence of only one copy of the *NF1* gene by applying either PRA analysis or/and SNP-array analysis. For the remaining four neurofibromas we were not able to determine the *NF1* copy number due to the lack of DNA (Supp. Table S2).

### Copy Neutral LOH Mechanisms

**Figure 4:**
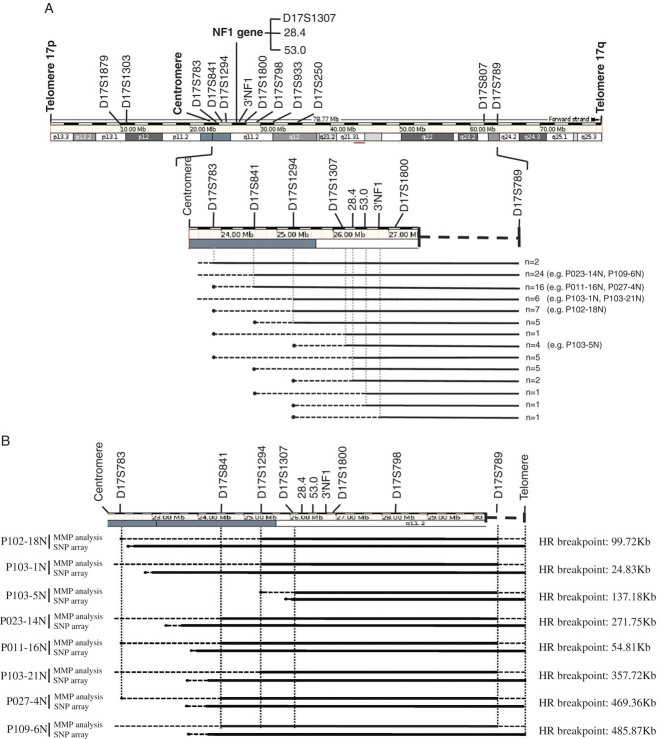
Mitotic recombination crossover mapping. **A:** Crossover analysis by Multiplex Microsatellite PCR (MMP). **B:** Mapping refinement by SNP-array compared to MMP. Solid black bar: uniparental isodisomy (B); dashed line: uncertain region; black circle: no uniparental isodisomy. HR, Homologous recombination. [Color figures can be viewed in the online issue, which is available at wileyonlinelibrary.com.]

We identified 80 dNFs exhibiting LOH that started somewhere between the centromere of chromosome 17 and the *NF1* gene and extended to the 17q telomere (Fig. 1C, Table 1, Supp. Table S2). Thus, the LOH detected in these tumors involved almost the entire long arm of chromosome 17. Importantly, two copies of the 17q arms were present in these tumors, as well as the two copies of the *NF1* gene carrying the constitutional mutation [Serra et al., 2001b]. The mechanism generating LOH in these tumors was homologous recombination (mitotic recombination) with a unique crossover between the centromere and the *NF1* gene, generating isodisomy from the crossover region up to the 17q telomere (as evidenced by SNP-array analysis) (Fig. 1C). This mutational mechanism was found to be the cause of ∼62% of all LOH events detected in dNFs (Table 1). Again, as for the fine mapping of deletion breakpoints, the use of microsatellites did not facilitate a precise location of recombination breakpoints (or crossovers) (Fig. 4A). Nevertheless, in eight dNFs we were able to locate the crossover region at a higher resolution using SNP-array analysis (Fig. 4B). We found that in two dNFs the crossover was centromeric to the D17S783 marker, clearly located closer than 1 Mb from the centromere. However, in addition, there were a fair number of dNFs (*N* = 29) homozygous (noninformative) for marker D17S783, for which the crossover could also be located close to the centromere. We found that this was the case of tumors P102-18N and P103-1N (Fig. 4B). For five dNFs there were no informative 17p microsatellite markers (three from the same patient). We were not able to rule out the possibility of a loss of the complete chromosome 17 with an endoreduplication of the remaining one, in these cases. There were also a significant number of dNFs for which the crossover was apparently located closer than 1 Mb from the *NF1* gene. In eight dNFs the breakpoint was clearly located between marker D17S1294 and the *NF1* gene. After performing SNP-array analysis we found that in tumor P103-5N the crossover was located in a region of 260–400 kb close to the *NF1* transcription start site. However, in many dNFs the crossover seemed to occur in a region equidistant from the centromere and the *NF1* gene (see Fig. 4B).

### Other Genomic Alterations in Neurofibromas

**Figure 5:**
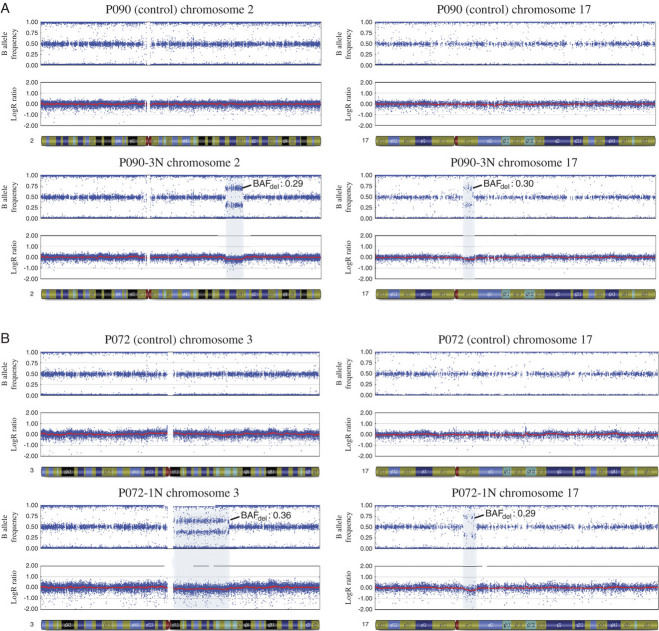
SNP-array analysis of samples P090-3N and P072-1N compared to their respective controls. **A:** Detection of deletions in 2q and the *NF1* region in tumor P090-3N; **B:** Detection of deletions in 3q and the *NF1* region in tumor P072-1N. Deletions were evidenced by a four-band pattern in the B allele frequency plot and a Log*R* ratio<0.

We performed SNP-array analysis in 19 dNFs to obtain a higher resolution on the LOH status of the *NF1* locus and in addition to scan their whole genome for the presence of additional copy number changes or copy neutral LOH. Seventeen dNFs exhibited no copy number changes or LOH elsewhere in the genome except for the 17q alteration involving the *NF1* gene. We identified two dNFs containing a large deletion: one located in chromosome 2q24.2–q31.1 (P090-3N) of about 15.3 Mb and the other in chromosome 3q11.2–q22.1 (P072-1N) of about 39.8Mb (Fig. 5). None of the deletions were present in the matching control DNAs, evidencing somatic events. In tumor P090-3N chromosome 2 and 17q-*NF1* deletions were present in the same proportion of cells (∼49%), suggesting that both alterations affected the same cells. In contrast, in tumor P072-1N, the 17q-*NF1* deletion was present in 49% of the cells while the chromosome 3q deletion was only present in ∼37% of the cells. This finding could reflect that either the 3q deletion occurred later during tumor development or that both deletions together do not confer a proliferation advantage to the cell.

### Variation in Neurofibroma-LOH Frequencies in Distinct NF1 Patients

Our final objective, in addition to have a more definitive picture of neurofibroma-LOH, was to have a cohort of NF1 patients with information about the phenotype at the molecular level, consisting of the characterization of LOH frequencies in dNFs and also the mechanisms responsible for the identified LOH. We calculated the minimal amount of dNFs needed to estimate the LOH prevalence in each patient, under the hypothesis of a finite number of dNFs, using different scenarios of precision and with a 95% confidence level (see Materials and Methods). For patients with 50 to 1,000 dNFs, we calculated that at least 10 dNFs had to be analyzed to estimate the LOH prevalence in these patients with a precision of 20% (see Supp. Fig. S1). Among the 113 patients, in 13 (∼10%) we studied at least 10 dNFs (Table 2). Although our results are still preliminary, the prevalence of LOH in dNFs in this group of 13 patients ranges from less than 10 to 50% or even higher. We also focused in the mechanism generating LOH. Most patients had dNFs with LOH caused by either deletions or recombination leading to copy neutral LOH. However, there were also patients with a clear dominant LOH mechanism. For example, dNFs of patient P082 exhibited mainly LOH caused by deletions, whereas dNFs of patients P022 and P078 had LOH primarily due to mitotic recombination (Table 2), regardless of LOH prevalence in dNFs. Two patients bearing the same germline *NF1* mutation (P062 and P082) exhibited a quite different prevalence of LOH in dNFs and a different preference of LOH-generating mechanism (Table 2).

**Table 2:** Interindividual Variation in Neurofibroma-LOH and the Mechanisms Responsible

						Germline mutation	
						
Patient	dNFs analyzed	dNFs with LOH	% LOH	% LOH-deletion (no. dNFs)	% LOH-MR (no. dNFs)	DNA level	Protein level
P011	14	5	35.7	20 (1)	80 (4)	c.3826C>T	p.Arg1276X
P020	14	2	14.3	0	100 (2)	c.2041C>T	p.Arg681X
P022	19	9	47.3	22.2 (2)	77.8 (7)	c.1756_1759delACTA	p.Thr586fsX18
P023	86	14	16.2	35.7 (5)	64.3 (9)	c.3525_3526delAA	p.Arg1176fsX18
P027	14	1	7.1	0	100 (1)	c.6226delG	p.Ala2076fsX14
P052	11	1	9.1	100 (1)	0	c.6791_6792dupA	[p.Tyr2264X; p.Ala2253_Lys2286del]
P055	11	1	9.1	0	100 (1)	c.5710G>T	p.Glu1904X
P062	16	6	37.5	33.3 (2)	66.7 (4)	c.910C>T	[p.Arg304X; p.Lys297_Lys354del]
P078	48	6	12.5	0	100 (6)	c.4537C>T	p.Arg1513X
P082	9	5	55.5	100 (5)	0	c.910C>T	[p.Arg304X; p.Lys297_Lys354del]
P084	10	2	20	0	100 (2)	c.4572C>G	p.Tyr1524X
P102	16	5	31.2	40 (2)	60 (3)	c.5242C>T	p.Arg1748X
P103	21	10	47.6	30 (3)	70 (7)	c.2338A>C	p.Thr780X
P104	11	3	27.3	0	100 (3)	c.4308G>T	p.Glu1436X

LOH, loss of heterozygosity; dNF, dermal neurofibroma; MR, mitotic recombination. Mutation numbering follows journal guidelines, with + 1 as the A of the initiation codon (codon 1) and based on *NF1* GenBank NM_000267.3.

## Discussion

A huge variability concerning the number of dNFs is observed in patients with NF1. Although genetic modifiers, unlinked to the *NF1* locus, are assumed to contribute to the variable number of dNFs [Easton et al., 1993; Sabbagh et al., 2009], the nature of these genes or genetic mechanisms are still unknown. With the long-term objective of identifying genes influencing the variation in number of dNFs, in the present study we have determined the prevalence of mechanisms generating LOH responsible for the inactivation of the *NF1* gene in these tumors. Our working hypothesis is that genes influencing the rate and efficiency of these mechanisms are key factors of neurofibroma development, because somatic inactivation of the *NF1* gene is a necessary and limiting step in dNF development. We focused our attention to those DNA repair systems involved in mechanisms leading to LOH, especially homologous recombination, but certainly other DNA repair mechanisms, will also be involved. We analyzed 518 dNFs from 113 patients and obtained the most comprehensive picture of LOH prevalence in dNFs published so far and also a comprehensive overview of the mechanisms underlying these LOH events.

We identified LOH in 25% of the dNFs studied (129/518). This number is in agreement with the average of the previously reported LOH prevalence of dNFs (∼20%) (reviewed in [Serra et al., 2007; Steinmann et al., 2009]). It has been shown that NF1 microdeletion patients do not exhibit LOH as a second hit in dermal neurofibromas [De Raedt et al., 2006a] by analyzing SNPs outside the microdeletion region. In the present study only 6 out of 518 neurofibromas (1.1%) belonged to NF1-microdeletion patients (*N* = 4). We have not identified any LOH in these six neurofibromas, confirming previous observations. We found that LOH was basically generated by two types of mechanisms: interstitial deletions and mitotic recombination. Approximately 38% of all LOH events detected were caused by deletions in 17q, whereas 62% were caused by mitotic recombination with a single crossover between the centromere and the *NF1* gene, and reducing the constitutional *NF1* mutation to homozygosity. This finding highlights the importance of copy neutral LOH generated by mitotic recombination regarding the inactivation of the *NF1* gene in the peripheral nervous system. Taking into account that we detected LOH in 25% of all dNFs, mitotic recombination could be responsible of ∼15% of all somatic *NF1* inactivation in dNFs. Furthermore, our present study in conjunction with the one previously performed by Steinmann et al. [2009] on plexiform neurofibromas, indicates that the prevalence of LOH and the mechanisms underlying LOH in dermal and plexiform neurofibromas are fairly similar, although both tumors differ considerably concerning their position, growth pattern and potential for malignant transformation.

In addition to the analysis of a global LOH prevalence and the underlying mechanisms in all dNFs studied, we also considered the prevalence and nature of LOH events in dNFs from single NF1 patients. To start describing the individual LOH prevalence, we summarized in Table 2 data from 14 patients from whom 10 or more dNFs were analyzed (except for 1 patient), the minimal number of dNFs per patient required. We consider that a larger number of patients with 10 or more dNFs analyzed need to be collected to make any assessment about the interindividual variation regarding dNF-LOH prevalence and underlying events, with the adequate statistical power. However, considering just a description of the results, in several patients we observed a major LOH-causing mechanism (either deletion or mitotic recombination). In the case there is a significant variation in individual LOH prevalence among NF1 patients, for those individuals with a high dNF-LOH prevalence together with a predominant LOH-mechanism, there exist the possibility that genes controlling the frequency of such mechanisms *in vivo* act as modifiers of the number of neurofibromas. For mitotic recombination these results would be in concordance with results obtained regarding the interindividual variation in mitotic recombination rates detected when analyzing the HLA-A locus in lymphocytes [Holt et al., 1999].

Most homologous recombination crossovers underlying LOH in neurofibromas were found to be located between the centromere and the *NF1* gene (Fig. 4). However, in a significant number of dNFs the LOH causing crossover events was likely to be close to the centromere; this would implicate chromosome and specifically centromere dynamics as the generators of double-strand breaks. On the other hand, the recombination crossovers of a few dNFs could be located really close to the *NF1* gene, even closer than in tumor P103-5N (Fig. 4B). For these cases it could be speculated that transcription-coupled repair may be implicated in the generation of crossovers. Mitotic recombination generating uniparental isodisomy in 17q has also been reported as the cause of *NF1* inactivation in NF1-associated myeloid malignancies [Stephens et al., 2006]. The authors postulated that in some leukemias isodisomies were generated by a double recombination with crossovers located in the centromere and the telomere of 17th chromosome. This mechanism, however, is unlikely to frequently cause LOH in dNFs. In all dNFs with LOH caused by mitotic recombination and analyzed by SNP-arrays (see Fig. 1C as an example), 17q isodisomy extended to the 17q telomere, with only one recombination crossover located between the centromere and the *NF1* gene. Finally, for five dNFs (three from the same patient) there were no informative 17p microsatellite markers raising the possibility of a loss of the complete chromosome 17 with an endoreduplication of the remaining chromosome. However, we believe this is not a very plausible possibility because this type of event has only been reported in the literature once for a dNF.

Somatic deletions in 17q were observed in 38% of the dNFs showing LOH (9.4% of the total number of dNFs included in our study). Deletions expanded roughly from ∼80 kb to ∼8 Mb. However, the large distance between microsatellite markers and the lack of heterozygosity in some cases make us to believe that MMP analysis is underestimating deletions equal or smaller to ∼100 kb. Elucidating the precise mechanism causing these interstitial deletions is an arduous task because it requires a proper characterization and sequencing of the deletion breakpoints. A higher resolution SNP-array analysis of these tumors could facilitate this work and help to dissect all the various possible mechanisms involved in generating these deletions.

Importantly, we identified one neurofibroma (P082-1N) bearing a type-2 *NF1* deletion, caused by a nonallelic homologous recombination (NAHR) between the *SUZ12* gene and its pseudogene, which are both located adjacent to the *NF1* low copy repeats. The breakpoint of the somatic deletion in tumor P082-1N was located in the *SUZ12* intron 4. This type of deletion has been also identified in patients with somatic mosaicism, with some cells carrying the deletion and others not carrying it. In these patients, however, the type-2 deletion was the first-hit mutation, which probably occurred during early embryonic development because it was found at a high percentage in blood lymphocytes [Steinmann et al., 2007]. The somatic type-2 deletion identified in tumor P082-1N in this study has to be considered as a second-hit mutation that leads to the loss of the wild-type *NF1* allele. Not all type 2 microdeletions can be PCR amplified by a primer set for common breakpoints, and thus we cannot rule out the involvement of NF1-REPs or the *SUZ12* sequences in the deletion identified in tumor P004-7N, because MLPA results were consistent with this type of deletion. Although it is clearly an infrequent mechanism, somatic NAHR involving the *SUZ* 12 sequences indeed leads to *NF1* loss in dNFs.

There have been different attempts to characterize the whole genome of dNFs in order to search for additional genetic alterations that could help to find other genes implicated in dNF development. However, although there is some disparity in different studies, reports using different methodologies, such us comparative genomic hybridisztion (CGH) [Mechtersheimer et al., 1999], cytogenetic karyotyping of dermal neurofibroma-derived Schwann cells [Wallace et al., 2000], cytogenetic karyotyping of short term neurofibroma cultures [Mertens et al., 2000], array-CGH [Mantripragada et al., 2008, 2009], Eric Legius, personal communication), and SNP-array analyses (Nancy Ratner and the NF1 Microarray Consortium, unpublished results), indicated a paucity of recurrent gross alterations in dNFs. SNP-array analysis of 19 dNFs allowed us to have a better idea of the global genome integrity of dNFs beyond the *NF1* region. We identified two dNFs with large deletions in two distinct chromosomal regions, one in 2q24.2–q31.1 (observed in tumor P090-3N) and the other in 3q11.2–q22.1 (in tumor P072-1N). These findings are consistent with the most recent findings obtained using array-CGH, where no gross alterations or just a few and sporadic copy number alterations were identified in dermal neurofibromas [Mantripragada et al., 2008, 2009]. The genomic alterations found in the present study appeared to be random events, as deduced from their low frequency and the lack of recurrence among different dNFs. It is unlikely that these somatic events are necessary for neurofibroma growth. Thus, the majority of discrete dermal neurofibromas do not frequently exhibit additional gross alteration in other genomic regions except for 17q. However, it remains to be investigated whether mutations such as small deletions, small insertions, and point mutations in genes or regulatory regions exist that would contribute to dNF growth. Nevertheless, the lack of any recurrent gross alteration in the genome of dNFs favors the idea that, genetically, *NF1* inactivation in neurofibroma-initiating cells (precursor cells that have the capacity to differentiate into Schwann cells) might be sufficient for dermal neurofibromas to develop (in an *NF1* (+/−) background). Recent results in a dermal neurofibroma mice model favors this view [Le et al., 2009].

The presence of instability in the analysis of microsatellite markers (MSI) in neurofibromas has been raised several times (see. i.e., [Ottini et al., 1995], or more recently [Thomas et al., 2010]). We have not found MSI in any of the 518 neurofibromas belonging to 113 patients regardless of their tumor burden, despite the fact that several of the markers used in the MMP analysis were previously tested to be useful for detecting MSI in pairs of colorectal tumors with MSI [Serra et al., 1997].

Taken together, our work provides a comprehensive overview of the somatic mutational mechanisms generating LOH in benign neurofibromas. This has implications when identifying possible modifiers that influence the rate and nature of somatic events leading to the inactivation of the *NF1* gene. These modifiers in turn could prove to influence the number of dNFs developed in NF1 patients.
