# Supplementary material for: Dissecting Loss of Heterozygosity (LOH) in Neurofibromatosis Type 1-Associated Neurofibromas: Importance of Copy Neutral LOH
Source: Hum Mutat. 2010 Oct 28;32(1):78–90. doi: 10.1002/humu.21387 (PMC3151547; doi:10.1002/humu.21387)
Supplement: Supplementary file 1 [file humu0032-0078-SD1.pdf]

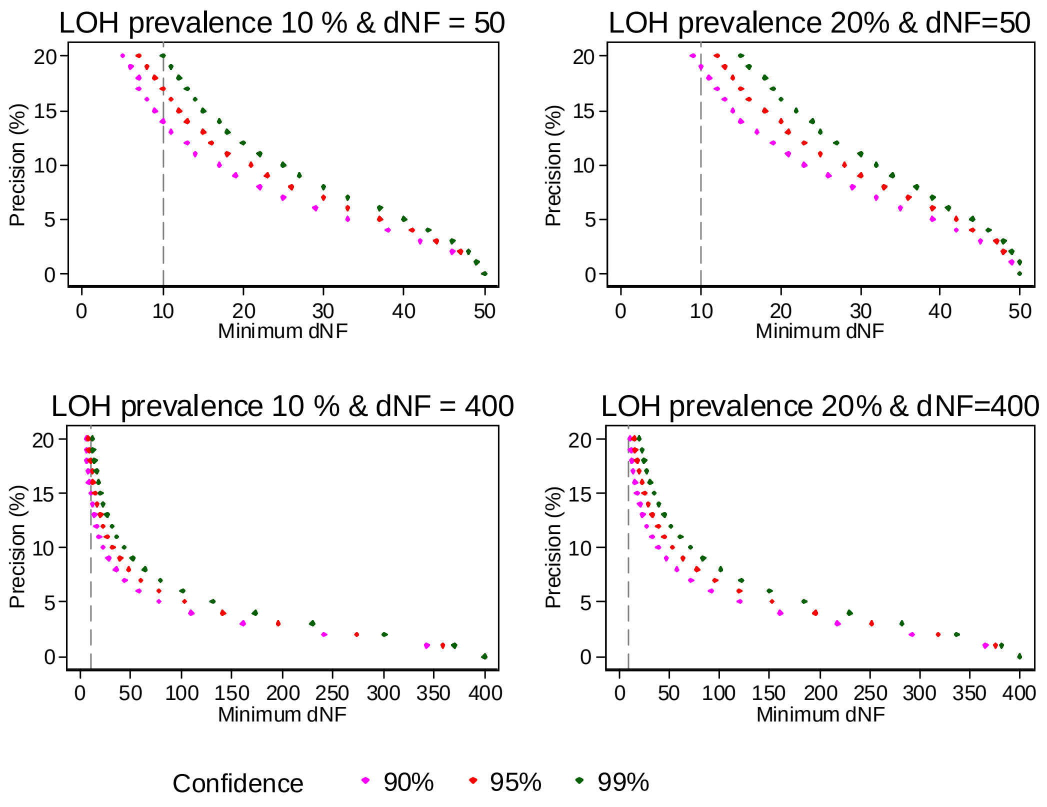

Supp. Figure S1. Precision expected in relation to the number of neurofibromas analyzed per patient and the confidence level, under different scenarios of: prevalence of dermal neurofibroma (dNF)-LOH and number of dNFs developed by a patient.

Supp. Figure S2

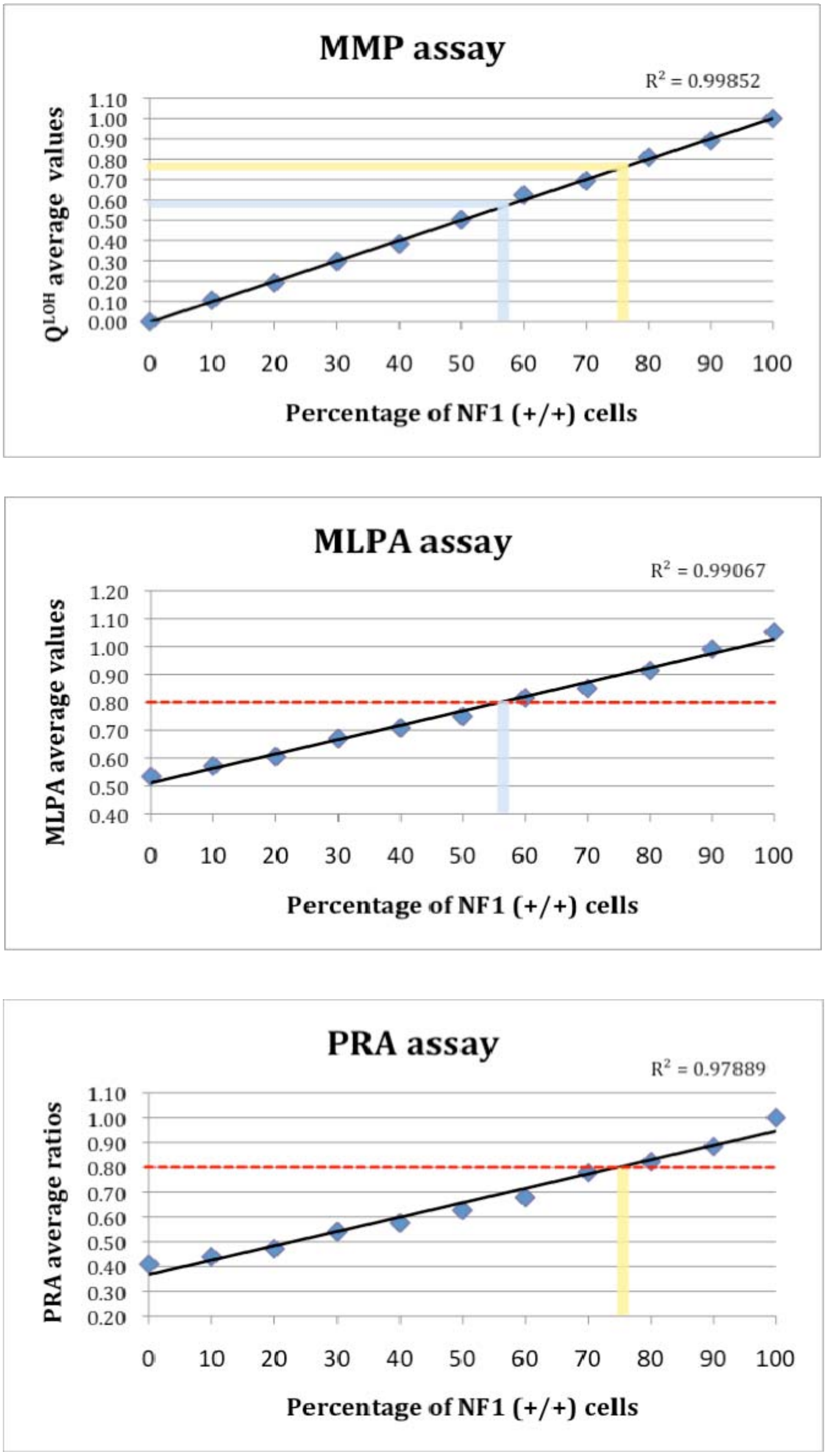

MMP assay

Percentage of normal cells (%)	Average Q^{LOH} values assay 1	Average Q^{LOH} values assay 2	Average Q^{LOH} values assay 3	Total average Q^{LOH} values	St. Deviation
100	1.00	1.00	1.00	1.00	0.0000
90	0.88	0.91	0.89	0.89	0.0178
80	0.83	0.82	0.78	0.81	0.0273
70	0.68	0.71	0.69	0.69	0.0130
60	0.59	0.65	x	0.62	0.0415
50	0.47	0.53	0.50	0.50	0.0313
40	0.37	0.40	0.38	0.38	0.0136
30	0.28	0.31	0.30	0.30	0.0112
20	0.18	0.19	0.20	0.19	0.0091
10	0.11	0.10	0.11	0.11	0.0024
0	0.00	0.00	0.00	0.00	0.0000

MLPA assay

Percentage of normal cells (%)	Average MLPA values assay 1	Average MLPA values assay 2	Average MLPA values assay 3	Total average MLPA values	St. Deviation
100	1.04	1.05	1.06	1.05	0.0122
90	0.98	0.98	1.01	0.99	0.0207
80	0.88	0.95	0.91	0.91	0.0335
70	0.80	0.86	0.89	0.85	0.0456
60	0.81	0.81	0.83	0.82	0.0100
50	0.72	0.74	0.78	0.75	0.0336
40	0.71	0.71	0.70	0.71	0.0068
30	0.68	0.66	0.67	0.67	0.0113
20	0.59	0.61	0.62	0.61	0.0142
10	0.57	0.57	0.58	0.57	0.0064
0	0.52	0.55	0.54	0.53	0.0135

PRA assay

Percentage of normal cells (%)	Average PRA values assay 1	Average PRA values assay 2	Average PRA values assay 3	Total average PRA values	St. Deviation
100	1.00	1.00	1.00	1.00	0.0000
90	0.90	0.88	0.87	0.88	0.0156
80	0.79	x	0.80	0.79	0.0035
70	0.76	0.82	0.76	0.78	0.0360
60	0.68	0.67	0.70	0.68	0.0175
50	0.62	0.63	0.63	0.63	0.0055
40	0.60	0.57	0.56	0.58	0.0225
30	0.56	0.52	x	0.54	0.0274
20	0.50	0.47	0.44	0.47	0.0278
10	0.42	0.46	0.43	0.43	0.0208
0	0.36	0.47	0.39	0.41	0.0610

Supp. Figure S2. We performed serial admixtures of two DNAs, one obtained from a patient carrying a constitutive deletion in the *NF1* gene (*NF1*+/-) and the other from its progenitor that did not transmitted the deletion (*NF1*+/+). These samples allowed us to reproduce hypothetical cellular composition of neurofibromas showing LOH due to the presence of a deletion in the *NF1* gene and carrying different proportions of “contaminating” normal cells. We created 11 samples with “contaminations” of NF1+/+ cells ranging from 0% (100% of *NF1*+/- cells) up to 100% (0% of *NF1*+/- cells) in

intervals incrementing “contamination” of NF1+/+ cells in 10%. With the same DNA admixtures we performed MMP, MLPA and PRA techniques in parallel, using conditions as described in Material and Methods section. For the three techniques, analyses were performed in triplicate (see Table attached to the Figure). These analyses allowed calculating the Q^{LOH} values (using MMP technique) for each of the different DNA admixtures and to establish a correlation between Q^{LOH} values and the limits of *NF1*-deletion detection by the MLPA and PRA techniques.

Supp. Figure S2 illustrates and compares the results obtained for the three techniques. Plotted in the X axes there are represented the different percentages of NF1+/- and NF1+/+ cells. Plotted in the Y axes there are represented: upper plot) for MMP analysis, the average of Q^{LOH} values obtained from at least 2 informative microsatellites located within the *NF1* gene; middle plot) for MLPA technique, the average normalized values obtained from *NF1* probes; lower plot) for the PRA analysis, the average normalized ratios obtained from the analysis of *NF1* gene/pseudogene.

As it can be seen in the upper plot, there is a good correlation between the Q^{LOH} value and the percentage of “contaminating” normal cells within a context of cells bearing a deletion in the *NF1* gene. By comparing the same admixtures of DNA using MLPA and PRA (middle and lower plots, respectively) we were able to establish the Q^{LOH} values that market the limit of detection for MLPA and PRA techniques. In the case of MLPA technique, the upper limit was a Q^{LOH} value of 0.58. In the case of PRA technique, the upper limit was a Q^{LOH} value of 0.76.

Dashed red line indicates the threshold value for considering the presence of an *NF1* deletion in the case of both techniques. Values below the red line (a normalized ratio of 0.8) indicate a copy loss in the *NF1* gene. Blue and yellow solid lines indicate the relationship between the limit of detection of an *NF1* deletion by MLPA and PRA techniques (respectively) and the Q^{LOH} value obtained in the MMP analysis.

MMP (Multiplex Microsatellite PCR); MLPA (Multiplex Ligation-dependent Probe Amplification); PRA (Paralog Ratio Analysis); R^2 (Coefficient of determination).

Supp. Table S1. Description of the primers used in the Microsatellite Multiplex PCR technique

Primer name	Primer sequence	Fragment length	Final concentration in MMP reaction (μM)	Dye
D2S2314	F- GGTGTCAGTGAGACCCTGT<br>R- ATTTCTAGCGGCCCTAAAAC	96-118	0.1	FAM
D17S1879	F- GAAGTTTACGAAAATTGCTGTC<br>R- AAGGGTAGTTCTGCGTGC	135-173	0.08	FAM
D17S1307	F- TAGGAGACCTGCTGCCTTT<br>R- AGGGCAGAGAAACCTAAGGA	209	0.05	FAM
D17S798	F- ACAACATTGTCAAACCCCATC<br>R- TGTTCTTGGGAGTGCAG	226-246	0.05	FAM
D17S1800	F- CTAAACTAGGTTGGGTTGAAATCTC<br>R- TCTGGCACAAAGACCTGAG	268-284	0.4	FAM
D17S250	F- GGAAGAATCAAATAGACAAT<br>R- GCTGGCCATATATATATTTAAACC	151	0.4	VIC
D17S933	F- ACTCACTGGGGTCCTGG<br>R- TGTGGTTTCCTTATAGACTGTAGA	187-206	0.08	VIC
D17S1303	F- CTCTCCAAGGCTCACTCAAA<br>R- TGGTCTTTTTCCATTCCAAA	225-245	0.05	VIC
D17S807	F- TCCACCTGTAGACCTGGTAAA<br>R- AGTGCTGCGTCTTACAACCT	114-138	0.6	NED
D17S789	F- ACTCCAAATCAAGTTTGTACTGAGA<br>R- CTGCATACGAAGGGTAGGAC	153-170	0.08	NED
D17S783	F- GATTTTCACCACTGAAACCA<br>R- CCTGGGGTAACAGAAAACTT	200-214	0.1	NED
D17S1294	F- TGGCATGCAATTGTAGTCTC<br>R- TTCTTTCCTTACTAAGTTGAGAACG	248-272	0.08	NED
53.0	F- CAGAGCAAGACCCTGTCT<br>R- CTCCTAACATTTATTAACCTTA	170-187	0.3	PET
28.4	F- GTTCTCAACTTAAATGTAAGT<br>R- GAACATTAACAACAAGTACC	207-219	0.3	PET
3'NF1	F- CTTCCATGGCTGCTAACATC<br>R- CCCTGTGGTGTAGTTCAACA	233-249	0.08	PET
D17S841	F- AGTTGGACTTTCTTACATGGC<br>R- AGAATCTCATATTAGGGGAAGG	275-295	0.08	PET

Supp. Table S2. Summary of LOH analysis in 129 neurofibromas showing LOH in the *NF1* gene and other 17q regions

			17p		17q			NF1 gene			17q										
	**Tumor**	**D2S2314**	**D17S1879**	**D17S1303**	**D17S783**	**D17S841**	**D17S1294**	**D17S1307**	**28.4**	**53.0**	**3'NF1**	**D17S1800**	**D17S798**	**D17S933**	**D17S250**	**D17S807**	**D17S789**	MMP Q^{LOH} average values	Nº of *NF1* copies detected by MLPA analysis	Nº of *NF1* copies detected by PRA analysis	Nº of *NF1* copies detected by SNP-array analysis	Mechanism leading to LOH
1	P001-1N																	0.29	Two copies			MR
2	P002-1N																	0.2	Two copies	Two copies		MR
3	P002-2N																	0.22		Two copies		MR
4	P004-5N																	0.46	One copy	One copy		Deletion
5	P004-7N																	0.27	One copy			Deletion
6	P007-1N																	0.35	Two copies			MR
7	P008-1N																	x				Deletion
8	P009-1N																	0.74				Deletion
9	P010-2N																	x				Deletion
10	P011-11N																	Pure		Two copies		MR
11	P011-16N																	0.52	Two copies		Two copies	MR
12	P011-4N																	0.37	Two copies	Two copies		MR
13	P011-7N																	0.43				Deletion
14	P011-9N																	0.32	Two copies	Two copies		MR
15	P016-2N																	x				Deletion
16	P018-1N																	x				Deletion
17	P018-3N																	x		One copy		Deletion (SSCP)
18	P020-2N																	x		Two copies		MR
19	P020-9N																	0.57		Two copies		MR
20	P022-11N																	0.26	Two copies	Two copies		MR
21	P022-15N																	0.28				MR
22	P022-17N																	0.6		One copy		Deletion
23	P022-19N																	0.33	Two copies			MR
24	P022-1N																	0.23	Two copies	Two copies		MR
25	P022-21N																	0.39	Two copies			MR
26	P022-2N																	0.41	One copy	One copy		Deletion
27	P022-6N																	0.26		Two copies		MR
28	P022-8N																	Pure	Two copies			MR
29	P023-14N																	0.29		Two copies	Two copies	MR
30	P023-16N																	x	Two copies	Two copies		MR
31	P023-21N																	0.33	Two copies	Two copies		MR
32	P023-25N																	0.27	Two copies	Two copies		MR
33	P023-38N																	x				Deletion
34	P023-42N																	0.25		Two copies		MR
35	P023-4N																	0.22	Two copies	Two copies		MR
36	P023-51N																	0.3	Two copies	Two copies		MR
37	P023-52N																	0.31	Two copies	Two copies		MR
38	P023-5N																	0.26	Two copies	Two copies		MR
39	P023-62N																	0.42	One copy			Deletion
40	P023-6N																	0.36	One copy	One copy	One copy	Deletion
41	P023-97N																	0.4		One copy	One copy	Deletion
42	P023-99N																	0.43	One copy			Deletion

43	P024-1N	x				MR
44	P027-4N	0.48	Two copies		Two copies	MR
45	P029-1N	x		One copy		Deletion (SSCP)
46	P029-2N	x		One copy		Deletion (SSCP)
47	P030-2N	0.26	Two copies			MR
48	P033-1N	0.52				Deletion
49	P034-1N	x	Two copies			MR
50	P034-4N	0.56		One copy		Deletion
51	P039-1N	0.48	One copy		One copy	Deletion
52	P040-1N	Pure	One copy			Deletion
53	P041-2N	Pure	Two copies			MR
54	P044-3N	0.57	Two copies			MR
55	P044-4N	0.56	One copy			Deletion
56	P045-1N	0.41	One copy			Deletion
57	P047-1N	0.37	Two copies			MR
58	P052-4N	0.52	One copy			Deletion
59	P054-1N	0.45		Two copies		MR
60	P055-2N	0.45		Two copies		MR
61	P056-1N	0.71		Two copies		MR
62	P058-10N	0.39	Two copies			MR
63	P058-7N	0.35	One copy			Deletion
64	P062-11N	0.35	One copy			Deletion
65	P062-1N	0.35	Two copies	Two copies		MR
66	P062-3N	0.42		Two copies		MR
67	P062-4N	Pure	One copy			Deletion
68	P062-6N	x		Two copies		MR
69	P062-8N	0.45	Two copies			MR
70	P065-1N	x	One copy			Deletion
71	P067-2N	Pure	One copy			Deletion
72	P068-2N	0.28	Two copies			MR
73	P068-3N	0.38	Two copies			MR
74	P072-1N	0.35	One copy		One copy	Deletion
75	P078-1N	0.32		Two copies		MR
76	P078-23N	0.3		Two copies		MR
77	P078-31N	0.36	Two copies	Two copies		MR
78	P078-34N	Pure		Two copies		MR
79	P078-39N	0.3		Two copies		MR
80	P078-45N	0.3	Two copies			MR
81	P079-1N	Pure	One copy		One copy	Deletion
82	P081-1N	0.55	One copy			Deletion
83	P081-2N	0.45	One copy			Deletion
84	P082-11N	0.66		One copy		Deletion
85	P082-12N	0.71		One copy	One copy	Deletion
86	P082-1N	0.53		One copy		Deletion
87	P082-6N	0.6		One copy	One copy	Deletion
88	P082-8N	x		One copy		Deletion
89	P084-2N	0.33	Two copies			MR
90	P084-5N	0.35		Two copies		MR
91	P085-2N	0.74	Two copies			MR
92	P087-3N	0.28	One copy			Deletion
93	P088-2N	x				Deletion
94	P089-3N	0.35	Two copies			MR

	Tumor	D2S2314	D17S1879	D17S1303	D17S783	D17S841	D17S1294	D17S1307	28.4	53.0	3'NF1	D17S1800	D17S798	D17S933	D17S250	D17S807	D17S789	MMP Q^{LOH} average values	Nº of *NF1* copies detected by MLPA analysis	Nº of *NF1* copies detected by PRA analysis	Nº of *NF1* copies detected by SNP-array analysis	Mechanism leading to LOH
95	P090-3N																	0.42	One copy		One copy	Deletion
96	P095-1N																	0.42	One copy		One copy	Deletion
97	P096-3N																	Pure	Two copies			MR
98	P096-5N																	Pure	One copy			Deletion
99	P097-1N																	x		Two copies		MR
100	P098-1N																	0.66		Two copies		MR
101	P102-18N																	0.31	Two copies	Two copies	Two copies	MR
102	P102-19N																	0.33	Two copies			MR
103	P102-3N																	0.44	One copy			Deletion
104	P102-4N																	0.71				Deletion
105	P102-5N																	0.43	Two copies			MR
106	P103-12N																	0.47		One copy		Deletion
107	P103-13N																	0.31		Two copies		MR
108	P013-14N																	0.63			One copy	Deletion
109	P103-15N																	0.58		One copy		Deletion
110	P103-1N																	0.35	Two copies	Two copies	Two copies	MR
111	P103-21N																	0.38	Two copies		Two copies	MR
112	P103-4N																	0.34		Two copies		MR
113	P103-5N																	0.34	Two copies	Two copies	Two copies	MR
114	P103-6N																	0.32	Two copies	Two copies		MR
115	P103-9N																	0.32	Two copies	Two copies		MR
116	P104-5N																	0.27		Two copies		MR
117	P104-7N																	0.45	Two copies	Two copies		MR
118	P104-8N																	0.29		Two copies		MR
119	P106-1N																	x				MR
120	P108-1N																	0.42	Two copies			MR
121	P109-1N																	0.35	Two copies			MR
122	P109-5N																	0.4	Two copies			MR
123	P109-6N																	0.32			Two copies	MR
124	P109-7N																	0.36	Two copies			MR
125	P112-4N																	0.43	Two copies			MR
126	P113-1N																	0.37	Two copies			MR
127	P113-2N																	0.33	Two copies			MR
128	P113-3N																	0.29	Two copies			MR
129	P113-4N																	0.3	Two copies			MR

Non LOH

LOH

Non informative

Mutational mechanism inferred

x Non MMP Q^{LOH} average value available

Supp. Table S2 legend: In colors it can be seen the presence, absence or non-heterozygous status of each microsatellite marker amplified in the multiplex PCR reaction. Extension of detected LOH can also be assessed. The subsequent columns describe the mean Q^{LOH} value (the average allele ratio calculated considering microsatellite markers D17S1307, 28.4, 53.0, 3'NF1 and D17S1800 when informative), the technique used for determining *NF1* copy number, and the LOH-mechanism.
